# Fundamental Insights on the Physical and Chemical Properties of Organosolv Lignin from Norway Spruce Bark

**DOI:** 10.1021/acs.biomac.2c00457

**Published:** 2022-07-11

**Authors:** Barbara Rietzler, Maria Karlsson, Isabella Kwan, Martin Lawoko, Monica Ek

**Affiliations:** Division of Wood Chemistry and Pulp Technology, Department Fibre and Polymer Technology and Wallenberg Wood Science Centre (WWSC), School of Engineering Sciences in Chemistry, Biotechnology and Health (CBH), KTH Royal Institute of Technology, Teknikringen 56, Stockholm SE-100 44, Sweden

## Abstract

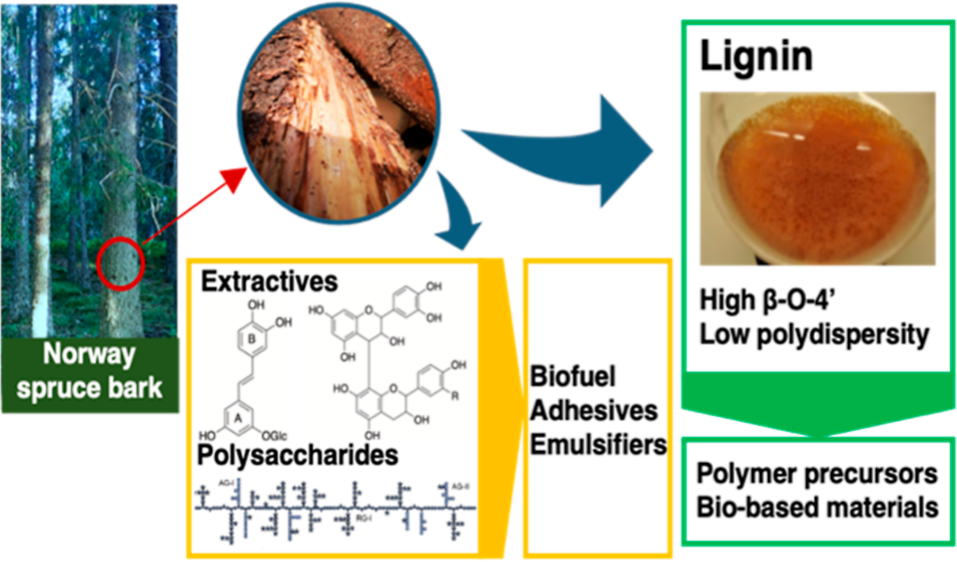

The interest in the bark and the attempt to add value to its utilization have increased over the last decade. By applying an integrated bark biorefinery approach, it is possible to investigate the recovery of compounds that can be used to develop green and sustainable alternatives to fossil-based materials. In this work, the focus is on extracting Norway spruce (*Picea abies*) bark lignin via organosolv extraction. Following the removal of the extractives and the subcritical water extraction to remove the polysaccharides, a novel cyclic organosolv extraction procedure was applied, which enabled the recovery of lignin with high quality and preserved structure. Main indicators for low degradation and preservation of the lignin structure were a high β-O-4′ content and low amounts of condensed structures. Furthermore, high purity and low polydispersity of the lignin were observed. Thus, the obtained lignin exhibits high potential for use in the direct development of polymer precursors and other bio-based materials. During the extraction sequence, around 70% of the bark was extracted. Besides the lignin, the extractives as well as pectic polysaccharides and hemicelluloses were recovered with only minor degradation, which could potentially be used for the production of biofuel or other high-value products such as emulsifiers or adhesives.

## Introduction

In respect of the increasing environmental concerns caused by the production of chemicals, energy, and materials from fossil fuels, the search for alternatives has gained attention over the last decade. The forest industry provides a large amount of available lignocellulosic biomass and thus a source of renewable materials to replace fossil-based materials.^1^ However, the increased interest in these materials requires efficient and optimal exploitation of the resources. Currently, large amounts of side products, such as bark and lignin, are generated in the timber, paper, and pulp industries in the Nordic countries, and these are mainly used in an inefficient way for energy production by combustion.^2^ The production of value-added materials, fuels, and chemicals from this biomass in an integrated biorefinery will provide full valorization while pursuing a zero-waste philosophy.^3^

One of the main side products generated by the forest industry is the tree bark. Due to its chemical richness, high availability, and low cost, it shows great potential for the production of value-added materials. To provide a more efficient use of this inexpensive raw material, integrated bark biorefinery concepts have been investigated.^4−8^ The composition of the bark can vary considerably compared to the respective wood; however, the main constituents are the same, namely, cellulose, hemicellulose, lignin, and extractives.^9,10^ Considering these compounds and their already known properties, their utilization for the production of high-value materials seems to be obvious. The most challenging aspect, though, is to separate the different components because the chemical richness also results in higher complexity. Therefore, it is very important to understand the structure and composition in detail. Only a detailed knowledge of the individual components will allow for the creation of a feasible strategy to generate an integrated biorefinery of the bark that can be applied on an industrial scale.

Several studies on the fundamental structure and composition of the bark of various tree species have been performed in recent years.^5,9−12^ One of the most abundant species and also readily available as a side product of the forest industry in large quantities is the Norway spruce bark. Its chemical composition and structure and the potential applications of the individual constituents have already been studied.^9^ Substantial research has been performed on the characterization of the hydrophilic extractives, condensed tannins, and stilbene glucosides, which have attracted great attention because of their bioactivity and antibacterial and antioxidant properties.^13−15^ Furthermore, the extraction and recovery of the polysaccharides as well as the isolation of nanocellulose from the bark have been examined, although in a smaller number of studies compared to the extractives.^13,16,17^ Hence, substantial work has to be done on the valorization of the bark.

Nonetheless, the fourth main component, lignin, has only been dealt with in a few studies. In general, lignin was already revealed to show high potential as a replacement for fossil-based materials.^18−21^ It is a complex, natural aromatic compound that is built from the three main hydroxycinnamyl alcohols, *p*-coumaryl, coniferyl, and sinapyl alcohol.^22^ Recently, a study on the fundamental structure of Norway spruce bark lignin has been published. By extracting milled bark lignin (MBL), it was shown that phenolic compounds, such as the hydroxy stilbene glucosides, are incorporated in the structure of Norway spruce bark lignin and behave as true lignin monomers.^23^ Yet, the structure of lignin depends strongly on the extraction method used to recover the lignin. The commonly recovered kraft and sulfite lignin from the pulping industry show a nonuniform structure and low purity, which limits their use for high-value application.^24^ An alternative extraction method is the organosolv process, where aqueous organic solvents such as alcohols or ketones are used in combination with organic or mineral acids as catalysts. The relatively mild conditions allow for the extraction of lignin with a more uniform structure and higher purity.^25^ However, economic aspects have so far prevented its application on an industrial scale. Thus, although the quality of the lignin is comparable or even superior to conventionally recovered lignin, these methods cannot be replaced yet.

Precisely, the extraction of lignin from the bark by organosolv extraction has been studied only in a few works. Organosolv lignin (OSL) has been extracted and characterized from sugar maple bark,^26^ pine and oak bark^27^ and willow bark.^28^ In all works, high purity of the lignin has been reported. In many cases, organosolv pulping is used for the production of biofuels from the resulting residue as well as the aqueous phase where the dissolved polysaccharides can be found. Thus, the organosolv process can be seen as a circular bioeconomy concept that facilitates the production of valuable biopolymers (lignin and cellulose) and advanced biofuels obtained from a low-cost byproduct of the forest industry. Environmental and cost assessment of this process show that there is potential for the OSL production process from the bark.^29^ The use of an inexpensive raw material from which high-value lignin is obtained could increase the economic feasibility of the process.

In this study, we hypothesize that high-quality lignin can be extracted from Norway spruce bark while also recovering the other individual constituents for value-added applications and biofuel production in a sustainable engineered concept. A sequence of Soxhlet extraction and subcritical water extraction in an accelerated solvent extractor (ASE) was followed by a mild organosolv extraction to recover the lignin with ethanol/water and 1.5 wt % sulfuric acid (H_2_SO_4_) as a catalyst. The subcritical water extraction has already been investigated and reported in a previous work.^30^ A novel cyclic extraction process^31^ was investigated for bark, which was expected to reduce degradation of lignin by the cyclic concept. Investigation of the extracted lignin by two-dimensional (2D) NMR and molecular weight analysis gave an insight into the fundamental structure of OSL from the bark with low degradation and will help to improve the process for future applications.

## Materials and Methods

### Materials

The bark of Norway spruce was sampled from a tree cut in Norra Djurgården (Stockholm, Sweden) in July 2020. The inner and outer barks were manually separated using a scalpel. The inner bark was freeze-dried and ground with a Wiley mill (3383L70, Thomas Scientific, New Jersey, USA) to a particle size of 20 mesh.

All chemicals were purchased from Sigma-Aldrich in analytical grade and used without further purification, unless stated otherwise.

### Sequential Extraction of Inner Bark

The ground inner bark was sequentially extracted starting with Soxhlet extraction in acetone/water (9:1 v/v, 40 cycles) to remove the extractives (hydroxy stilbene glucoside, and condensed tannins). The acetone was evaporated under reduced pressure, and the extractives were obtained in a powder form. Then, subcritical water extraction with an ASE (Dionex, California, USA) was performed at 100, 140, and 160 °C and a pressure of 100 bar. An extraction program with three cycles of 20 min was used at each temperature. The fractions were freeze-dried before further analysis.

### Lignin Extraction

After subcritical water extraction, the extraction of lignin with the solvent ethanol/water (70:30, v/v, 1.5 wt % H_2_SO_4_) at 160 °C and 100 bar was also performed using an ASE. The extraction was carried out in 15 cycles for 5 min each. MilliQ water was added to the extract to precipitate the lignin, and ethanol was removed by rotary evaporation under reduced pressure. The aqueous solution containing the precipitated lignin was centrifuged at 4688 G for 30 min in a Rotina 420 (Hettich Zentrifugen, Tuttlingen, Germany) instrument, and the precipitate was washed with MilliQ water two times. The obtained lignin was lyophilized before further analysis. The supernatant after precipitation and centrifugation was neutralized using sodium hydroxide solution 1 M, and the liquid was evaporated in a rotavapor, and complete dryness was reached after 3 h of drying in an oven at 60 °C. The bark residue after all ASE extractions was lyophilized.

### MBL

Acetone extracted bark (AEB) and the bark residue after all extractions [organosolv residue (OSR)] were ball-milled in a Retsch PM-400 planetary ball mill (Duesseldorf, Germany). The samples (ca. 8 g) were placed in 250 mL stainless-steel jars together with 40 grinding balls. The samples were milled for 24 h at 300 rpm in 1 h intervals with 30 min breaks. The ball-milled samples were extracted following the classical procedure.^32^ 5 g of sample was mixed with 125 mL of 96% (v/v) aqueous dioxane (25 mL solvent/g ball-milled bark). The mixture was stirred for 72 h at room temperature. The obtained lignin after centrifugation was freeze-dried, and no further purification was carried out.

### Carbohydrate Analysis

Carbohydrate composition of the samples was determined by acid hydrolysis in accordance with the TAPPI T222 om-06 standard.^33^ 200 mg of sample was mixed with 3 mL of 72% H_2_SO_4_ and placed under vacuum for 80 min with occasional stirring. MilliQ water (84 mL) was then added, and the hydrolysis was completed in an autoclave 2540EL (Tuttnauer Europe, Breda, The Netherlands) at 125 °C for 60 min. The mixtures were filtered on glass fiber filters to determine the Klason lignin, and the hydrolysate was further diluted for analysis with high-performance anion-exchange chromatography with pulsed-amperometric detection using an ICS 3000 system (Dionex, Thermo Scientific, California, USA).

### Size Exclusion Analysis

The samples were acetylated according to the protocol by Gellerstedt,^34^ dissolved in tetrahydrofuran (THF; 2 mg/mL), and filtered through a 0.45 μm PTFE syringe filter before injection. The separation was carried out on a Waters system (Waters Sverige AB, Sollentuna, Sweden) using Waters Ultrastyragel HR4, HR2, and HR0.5 (4.6 × 300 mm) solvent-efficient analytical columns in series with a Styragel guard column (THF, 4.6 × 300 mm) coupled to a Waters-2998 photodiode array detector operated at 254 and 280 nm and an RI detector. Calibration was performed with polystyrene standard solutions with molecular weights ranging from 176 kDa to 266 Da using the UV detector 254 nm channel. THF (HPLC grade) was used as the eluent.

### 1D and 2D NMR Analyses

All nuclear magnetic resonance (NMR) analyses were performed with a Bruker Avance III HD 400 MHz instrument (Bruker Corporation, Massachusetts, USA) at 300 K with a BBFO probe equipped with a *Z*-gradient coil. Sensitivity was improved with the gradient (e/a TAPPI). For the heteronuclear single quantum coherence (HSQC) experiments, 80 mg of sample was dissolved in 550 μL of deuterated dimethyl sulfoxide (DMSO). HSQC experiments were carried out with the “hsqcetgpsi” pulse program. A relaxation delay of 1.5 s was selected with a minimum of 80 scans using 1024 × 256 increments with additional 16 dummy scans. The spectra were processed with MestreNova software (version 9.0.0, Mestrelab Research). The spectra were Fourier transformed, and a baseline and manual phase correction was applied to both dimensions. The DMSO peak at δ_C_/δ_H_ 39.5/2.50 ppm was used as the internal reference. For the quantification, the signals of C2/H2 in lignin were selected as the internal standard.

^31^P NMR samples were prepared following the protocol reported by Argyropoulos et al.^35^ In short, 30 mg of lyophilized sample was dissolved in 100 mL of *N*,*N*-dimethylformamide and 100 mL of pyridine and phosphorylated with 2-chloro-4,4,5,5-tetramethyl-1,3,2-dioxaphospholane. The internal standard was endo-*N*-hydroxy-5-norbornene-2,3-dicarboximide.

## Results and Discussion

**Figure 1:**
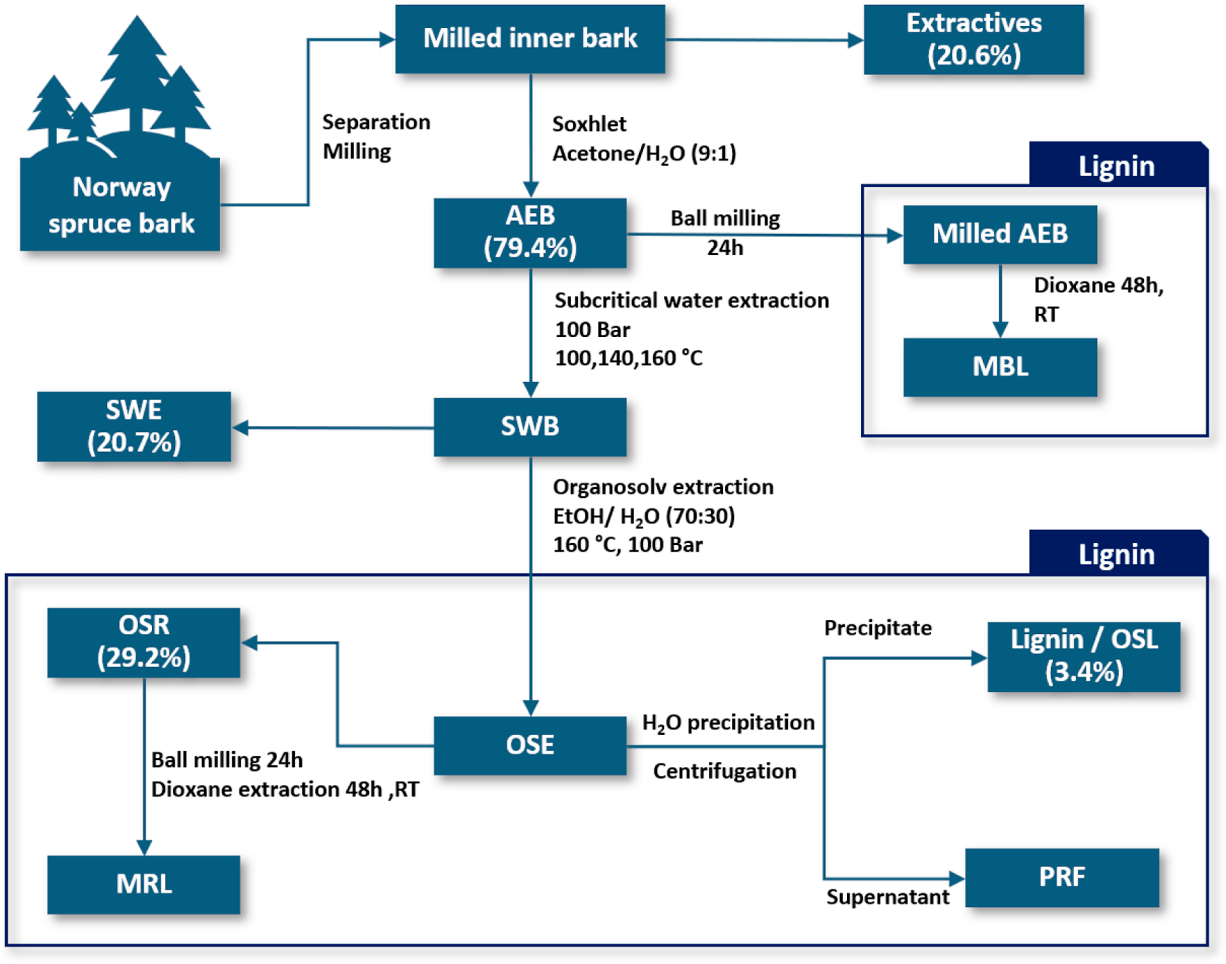
Schematic depiction of the extraction sequence of Norway spruce bark; AEB—acetone extracted bark, SWB—subcritical water extracted bark, SWE—subcritical water extracts, MBL—milled bark lignin, OSE—organosolv extract, OSR—organosolv residue, MRL—milled residue lignin, OSL—organosolv lignin, PRF—polysaccharide-rich fraction. The percentage represents the yield of the fraction in wt %.

In order to investigate and get a deeper insight into the structure of Norway spruce bark lignin while recovering other constituents, a mild extraction sequence was applied, as shown in Figure 1. Starting from the whole bark, separation into inner and outer bark was done to reduce the complexity and focus on the investigation of the inner bark. The sequential extractions were chosen based on previous studies.^17,30^

Soxhlet extraction with acetone/H_2_O (9:1, v/v) is commonly used to remove the hydrophilic and lipophilic compounds such as tannins, stilbene glucoside, and fatty acids. The removal of these components enables a more efficient extraction of the polysaccharides. The use of a Soxhlet apparatus reduces the amount of solvent that is required because of the cyclic extraction process, and the low temperatures facilitate mild extraction conditions.

Subcritical water extraction was selected to recover the polysaccharides from the bark with good yield and low degradation. At three temperatures 100, 140, and 160 °C, different fractions of polysaccharides were extracted. At 100 °C, some residual extractives, oligosaccharides, as well as some low-molecular-weight pectins were extracted. At 140 °C the fraction contained mainly pectic polysaccharides, whereas at 160 °C, hemicelluloses were extracted.

In order to extract and recover bark lignin within this extraction sequence, an organosolv extraction process was applied following the subcritical water extraction. In this study, a temperature of 160 °C, a solvent composition of EtOH/H_2_O (70:30, v/v), and 1.5 wt % sulfuric acid as a catalyst were selected. The extraction was performed directly after the subcritical water extraction, and the liquid–solid ratio (L/S) was kept at around 9. By using an ASE, a dynamic cyclic extraction was possible to be applied, which enables very mild extraction conditions by physical protection of the sensitive lignin bonds.

### Cyclic Organosolv Extraction Process

The organosolv process has been investigated in several studies regarding the recovery of lignin from wood; however, only a limited number of studies have investigated the extraction of lignin from bark. In the following, the focus is mainly on the properties of lignin extracted by a cyclic organosolv extraction process. The cyclic organosolv process has previously been developed by Karlsson et al.,^31^ where an additive-free process using physical protection to preserve the lignin structure was designed. Within this study, the mildness of the extraction process as well as the lignin properties of Norway spruce inner bark lignin are studied.

The β-O-4′ content was used as an indication for mild extraction conditions and low degradation of the lignin. The amount of β-O-4′ linkages and other native linkages in lignin were determined by semiquantitative 2D HSQC NMR analysis (Table 1). By integration of the β-O-4′ Cα position (hydroxylated and etherified), an amount of 30% was determined and was similar to that found in the extracted wood lignin by the cyclic organosolv process.^31^ In general, organosolv processes are known to degrade the lignin, and the recovered lignin usually shows low β-O-4′ content. For example, Liu et al. extracted pine bark using an organosolv one-batch process and obtained a significantly lower β-O-4′ content compared to dioxane-extracted lignin from the pine bark.^27^ It is presumed that the cyclic extraction process provides physical protection of the labile β-O-4′ bonds, and therefore they are not cleaved as much as in the one-batch process. This is because the dissolved material is removed from the reaction vessel before it reacts significantly. Second, replacement of the removed volume ensures a dilution of solutes, limiting the collisions necessary for condensation reactions.

**Table 1:** Lignin Interunit Linkage Type Contents from Integration of 2D HSQC NMR Spectra of Lignin Extracted from Norway Spruce Inner Bark^a^

	OSL	MBL	MRL
β-O-4′ aryl ethers	30 ± 2	31	17
β-5′phenylcoumarans	12 ± 1	12	6.2
β–β′resinols	1.9 ± 0.2	2.2	1.9
5-5′ dibenzodioxocins (DBDO)	0.5 ± 0.2	3.8	0.1
enolether	1.3 ± 0.1	1.0	
coumaraldehyde	2.3 ± 0.2	0.8	
Hibbert’s ketone	2.0 ± 0.1	1.2	2.1
Stilbene-β1	3.5 ± 0.5	1.4	1.1
Stilbene-β5	3.0 ± 0.3	0.4	1.5

a The results of the OSL linkage values are the mean of four measurements and the corresponding standard deviation. The standard deviation also applies for the measurement of MBL and MRL; OSL—lignin extracted by organosolv extraction, MBL—milled raw bark extracted with dioxane, and MRL—milled residue after organosolv treatment extracted with dioxane. The interunit linkages are semiquantified per 100 Ar units using the C2 region in the aromatic region as an internal standard.

The β-O-4′ content for spruce bark lignin extracted by dioxane in this study was found to be around 31 per 100 Ar units (**MBL**, Table 1). This value has rarely been reported before in the literature; only Neiva et al. reported a value of 44% based on total lignin interunit linkages.^18^ Thus, the OSL extracted by the cyclic process shows merely a slight reduction of β-O-4′ linkages compared to the MBL, which is an indication for very low degradation.

**Figure 2:**
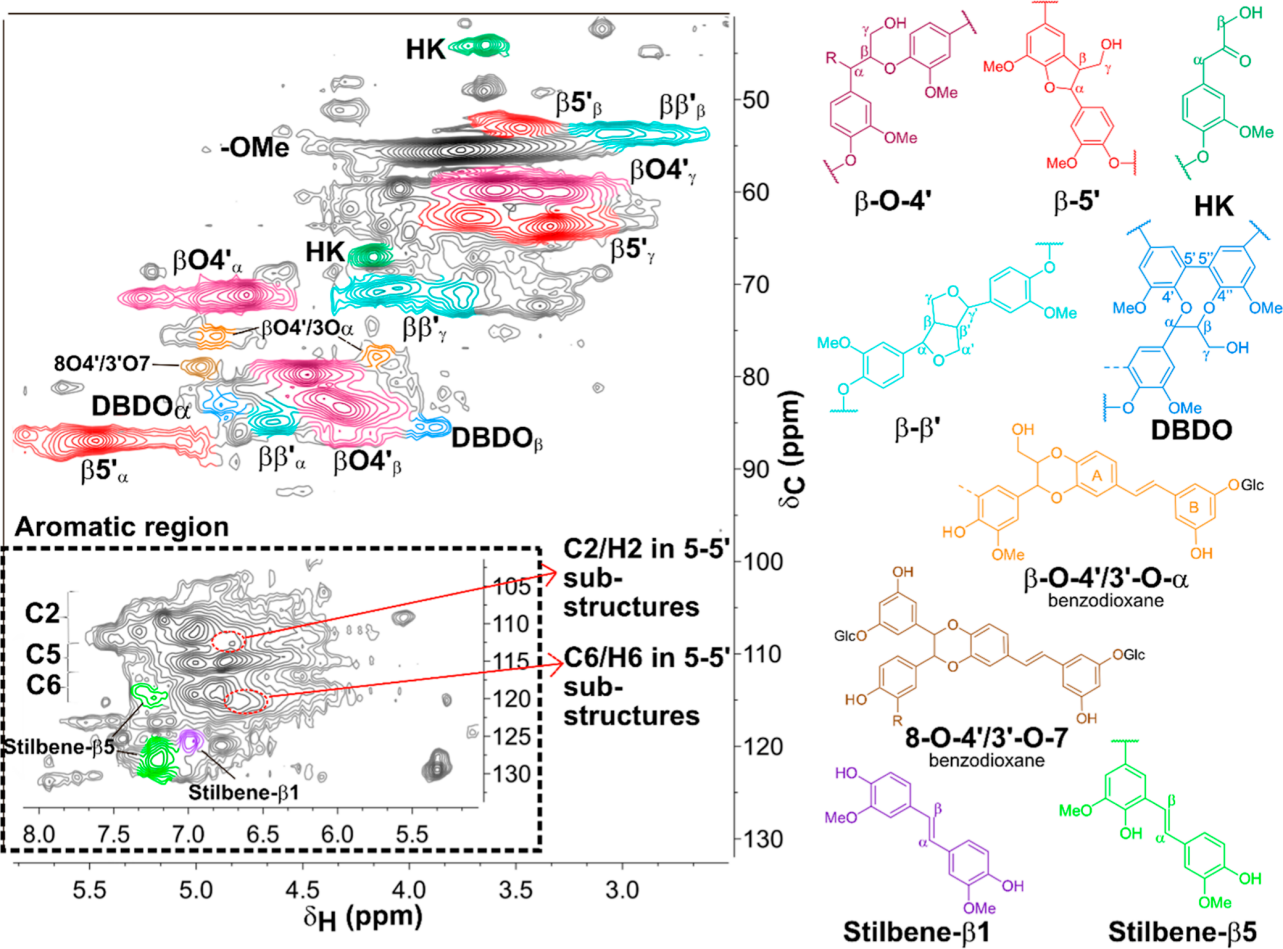
2D HSQC NMR of lignin extracted by cyclic organosolv process from Norway spruce inner bark in DMSO-*d*6. The main lignin interunit linkages are depicted to the right of the spectrum. **β-O-4′** aryl ethers; **β-5′** phenylcoumarans; **β–β′** resinols; **5-5′** dibenzodioxocins (**DBDO**); Hibbert’s ketone (**HK**); **β-O-4’/3-O-α** benzodioxane structure formed through coupling of coniferyl alcohol and astringin; **8-O-4’/3′-O-7** benzodioxane structure formed by stilbene glucoside units; Stilbene-**β1’**; Stilbene-**β5’.** Note that the glucoside units of the hydroxystilbene glucosides have been cleaved under the organosolv conditions.

It is observed for the extracted lignin that the β-O-4′ Cα position appears to be both hydroxylated and etherified. The signal of the β-O-4′ Cα etherified is observed at δ_C_/δ_H_ 79.7/4.5 ppm in the 2D HSQC NMR spectra (see Figure 2), and it is estimated by integration that around 57% of the β-O-4′ linkages are etherified, which is in accordance with the previously reported results from organosolv extracted lignin from spruce wood.^31^ The etherification of the Cα position happens through a nucleophilic addition reaction of ethanol to the benzylic cations formed under acidic conditions.^36^ Etherification of the β-O-4′ linkages increases the solubility of the lignin in ethanol. However, the etherification does not prevent cleavage of the β-O-4′ linkages, which is mainly avoided by the cyclic extraction process. Hence, the cyclic approach in this study facilitates the extraction of lignin with a preserved structure from Norway spruce inner bark and reduces the degradation of the lignin usually occurring in the organosolv process.

**Figure 3:**
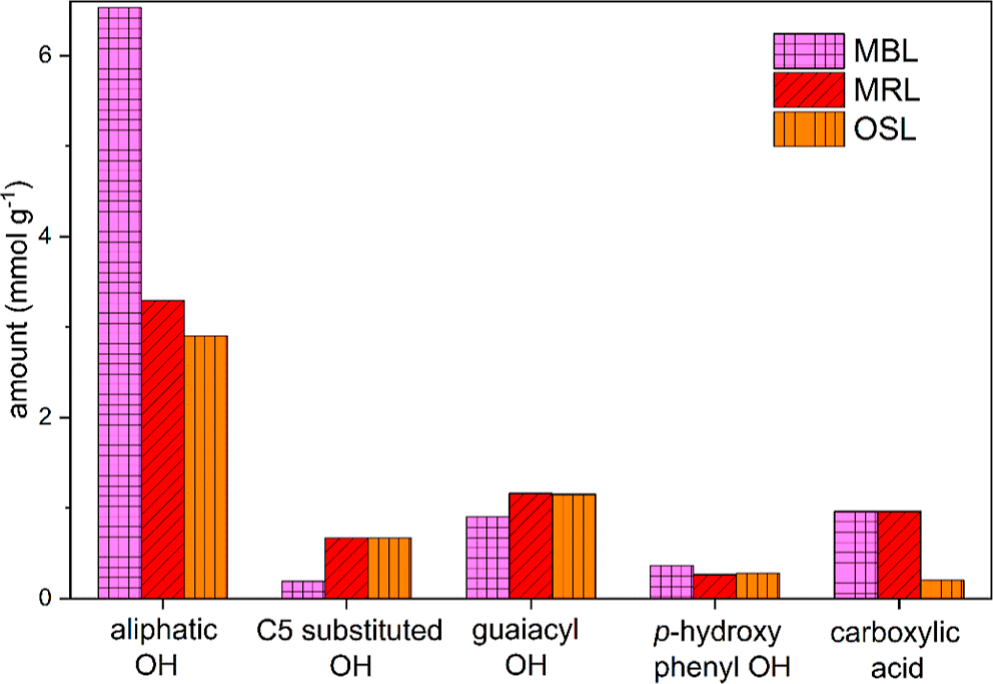
Comparison of hydroxy functionalities determined by ^31^P NMR between OSL, MBL, and MRL; the values are the mean of at least two measurements; the standard deviation was only determined for OSL.

The amount of typical lignin interunit linkages such as β-O-4′, β-5′, and β–β′ detected in the **OSL** compared to the milled raw bark lignin (**MBL**) are shown in Table 1. The 2D HSQC NMR spectra of MBL are shown in Figure S1 in the Supporting Information. It can be seen that the values are in the same range, and they are also in accordance with the values reported for cyclic-extracted OSL from spruce wood; however, the values reported in the literature for milled raw bark lignin from spruce bark are marginally higher. Some minor signals in the 2D HSQC NMR were observed at around δ_C_/δ_H_ 112.5/6.75 ppm and δ_C_/δ_H_ 120.5/6.60 ppm, which can be assigned to C2/H2 and C6/H6 of 5-5′ condensed substructures. Although the signals are still weak, compared to the MBL, they are slightly stronger. These 5-5′ linkages are both native and could be formed.^31,37^ It can be assumed that during the organosolv extraction, some of the β-O-4′ linkages are cleaved, which leads to the formation of β- and phenoxy radicals. From the phenoxy radicals, C5 radicals emerge, which then couple to the 5-5′ condensed substructures. Also, by quantitative ^31^P NMR analysis, it was determined that the amount of C5-substituted OH groups was higher compared to the MBL (Figure 3).

In another technical lignin, for example, in softwood kraft lignin, an amount of 0.9–1.3 mmol g^–1^ C5-substituted OH functionality is reported, which is considerably higher.^38^

Hibbert’s ketone are formed as minor products through an acidolysis reaction of aryl ether bonds. In summary, the OSL seems to be slightly more condensed and degraded compared to the MBL, but many indicators reveal mild extraction conditions with a perseverance of the lignin structure. Besides, the OSL shows less degradation compared to the other technical lignin.

Additionally, from the 2D HSQC NMR analysis, it can be said that the OSL from the bark has high purity since signals assigned to polysaccharides are only detected at trace levels. However, there are signals observed that suggest the presence of incorporated stilbene glucoside structures in the bark lignin, which has been reported recently on MBL by Rencoret et al.^23^ For example, by 8-O-4′coupling of an isorhapontin and an astringin unit, benzodioxane structures could be formed that would result in a peak at around δ_C_/δ_H_ 79.0/5.0 ppm, which was observed in the 2D HSQC NMR spectra of the OSL from the spruce bark (Figure 2). Similar signals have been reported for dimeric hydroxy stilbene structures detected in the extracts of Norway spruce bark, but the presence of dimeric hydroxy stilbene structures can be excluded since these structures are highly soluble in water; thus they are removed by the intensive extraction sequence.^39^ Therefore, it was proposed by Rencoret et al. that these hydroxy stilbene glucosides are incorporated in the lignin structure.^23^ Further evidence was provided by the detection of signals at δ_C_/δ_H_ 75.8/4.90 ppm and δ_C_/δ_H_ 78.0/4.15 ppm, which are assumed to result from the correlation of C_α_/H_α_ and C_β_/H_β_ of the formed benzodioxane structure by the cross-coupling of coniferyl alcohol and the catechol moiety of astringin (Figure 2). From the presence of these signals in the OSL, it can be speculated that the organosolv extraction is not cleaving these linkages either, and it is possible to extract lignin with incorporated stilbene structures by organosolv treatment. A significant difference compared to the previous work on MBL, however, is that in the OSL, the presence of glucose from the glucoside residue is very low. Thus, it is assumed that the linkage between the stilbene incorporated in the lignin and the glucoside residue is cleaved during the organosolv extraction through hydrolysis. The integration of stilbene glucosides into the lignin structure would provide the lignin with exceptional properties, such as antibacterial and antioxidant properties. The possibility to extract lignin with these additional structure types increases the potential for high-value applications.

Furthermore, the presence of stilbene structures formed as a result of the extraction process was detected by 2D HSQC NMR. Stilbene-β1′ and stilbene-β5′ are formed during the elimination of formaldehyde from phenylcoumaran (β5′) and spirodienone (β1′) (mechanism can be found in Scheme S1 in the Supporting Information). It can be clearly seen that those structures are formed during the extraction since their amount is significantly higher in the OSL than in the MBL. The signals of Stilbene-β1′ and stilbene-β5′ occur in the aromatic region at δ_C_/δ_H_ 120-130/6.9–7.4 ppm, which can be seen in Figure 2. In comparison, in the spectrum of MBL in Figure S1, the signals of the stilbene glucosides have a lower shift of δ_C_/δ_H_ 110—120/6.2—6.8 ppm.

**Figure 4:**
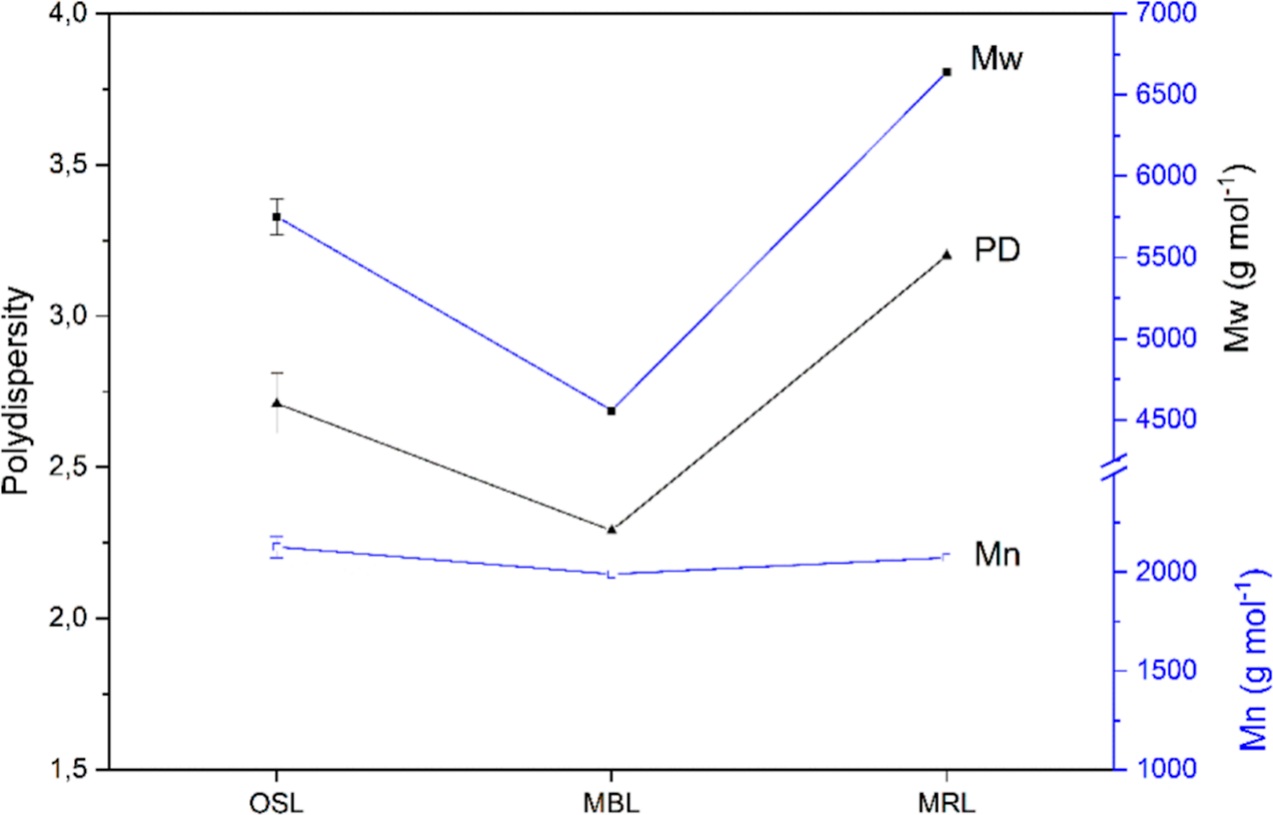
Weight-average (*M*_w_) and number-average (*M*_n_) molecular weights and corresponding PD of the OSL, the MBL, and the MRL determined by THF SEC; the values are the mean of at least two measurements; the standard deviation was only determined for OSL. Chemical composition before and after organosolv extraction.

The OSL was further analyzed by size exclusion chromatography (SEC) analysis to determine the molecular weight and polydispersity (PD). The number- and weight-average molecular weights as well as the PD are shown in Figure 4. As can be seen, the number-average molecular weight (*M*_n_) does not change significantly between the OSL and the MBL; however, the weight-average molecular weight (*M*_w_) seems to be higher in the OSL. The differences in *M*_w_ might be due to the fact that some of the low-molecular-weight lignin is already extracted during the subcritical water extraction; thus the OSL contains lignin with higher molecular weight. Also, the MBL was extracted with dioxane, and no further purification to remove polysaccharides was performed; therefore, the MBL contains a significant amount of low-molecular-weight polysaccharides.

*M*_n_, however, is considered to be the more representative value when it comes to molecular weight determination of lignin. The ball milling of the bark before dioxane extraction to obtain the MBL and MRL might also have an effect on the molecular weight of the lignin since it might cleave some interunit linkages; therefore, it does not represent the true molecular weight before ball milling. It can be seen that the PD of the OSL is low, which is especially preferred when it comes to the application of lignin as polymer precursors.^40,41^

**Figure 5:**
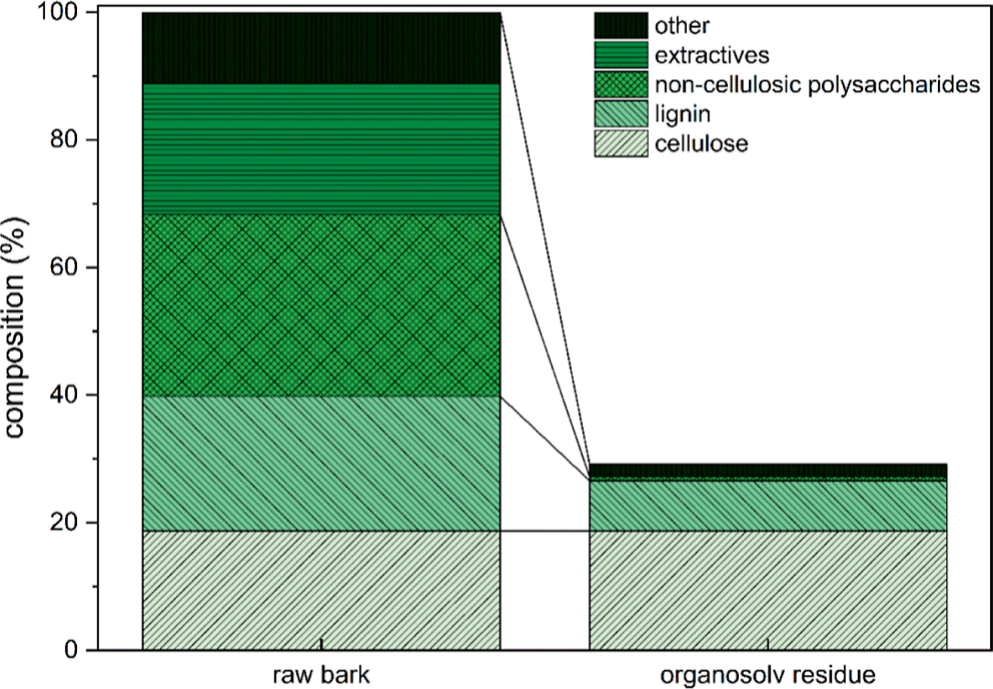
Chemical composition of Norway spruce bark before and after organosolv extraction.

To determine the efficiency of the extraction sequence, the chemical composition of the raw bark and the residue after organosolv extraction was analyzed by acid hydrolysis. In the beginning, the raw inner bark is composed of 20.6% extractives, 28.5% noncellulosic polysaccharides, 18.7% cellulose, 21.1% lignin, and 11.1% other minor components; the exact composition can be found in Table S1 in the Supporting Information. As shown in Figure 5, 70.8% of the bark is extracted within the extraction sequence, and the obtained residue is rich in cellulose, as well as some remaining lignin. The residue contains 18.7% cellulose, 7.9% lignin, 0.6% noncellulosic polysaccharides, and 2.0% other minor components based on the initial mass of the raw bark. Thus, most of the noncellulosic polysaccharides are removed during the extraction; the cellulose is preserved and around 63% of the lignin is removed, although in the organosolv extraction itself, only 3.4% lignin (based on initial raw bark mass) representing 16.1% of the total lignin is recovered. Therefore, further analysis of the subcritical water extracts was performed.

From 2D HSQC NMR, it can be concluded that some lignin is extracted during the subcritical water extraction since signals occurring in the aromatic region (δ_C_/δ_H_ 100-140/5-8 ppm) are observed in all spectra of the subcritical water extracts (Figure S2). These signals can be assigned to C2/H2, C5/H5, and C6/H6 of the G units of the bark lignin. Nevertheless, the intensity of the signals was low compared to the signals resulting from the carbohydrates present in the extract. The 100 °C fraction also showed some characteristic signals of the aromatic ring of the stilbene glucoside at δ_C_/δ_H_ 102.5/6.35 ppm C4/H4, δ_C_/δ_H_ 104.5/6.75 ppm C2/H2, and δ_C_/δ_H_ 106.9/6.55 ppm C6/H6. Therefore, the strong glucose and especially the C1/H1 signal around δ_C_/δ_H_ 100.4/4.8 ppm can be assigned to the glucoside residue. Thus, not all extractives were removed during the acetone/H_2_O extraction in the first step. Still some signals from rhamnose and galactose, which indicate the extraction of some pectic polysaccharides, are detected. On the contrary, the 140 °C fraction shows mainly signals resulting from correlations that can be assigned to pectic polysaccharides, such as rhamnose, arabinose, galactose, and galacturonic acid. A more detailed analysis of the pectin present in the Norway spruce bark has recently been reported by Le Normand et al.^42^ In the 160 °C fraction, signals of xylose and mannose in addition to the pectic sugar signals were observed. It was observed from 2D NMR HSQC that the polysaccharides found in all three extracts show almost no degradation. The pectin obtained from the extraction could therefore be used in higher-value applications, for example, as emulsifier or thickener in food applications.

**Figure 6:**
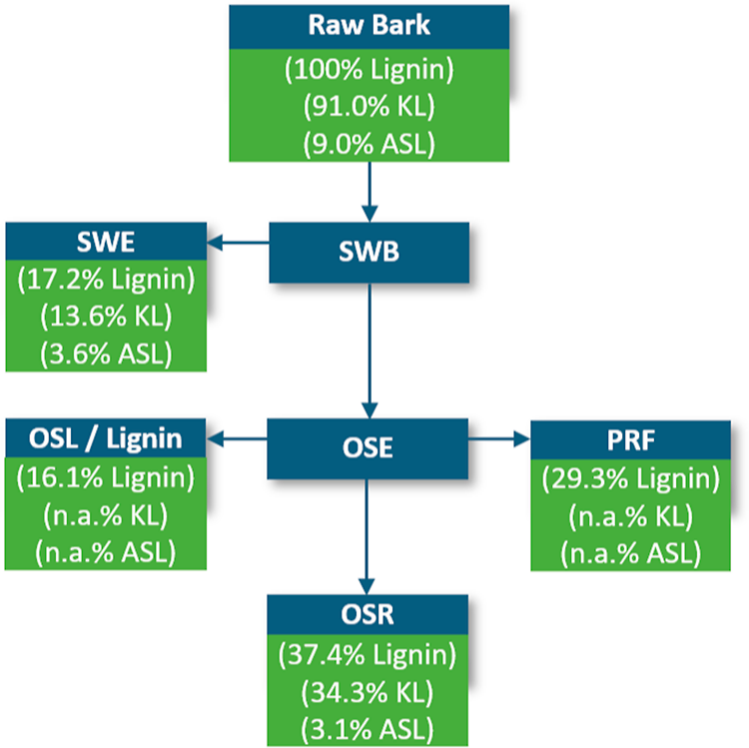
Illustration of each extraction step and the amount of lignin extracted in each; SWB—subcritical water extracted bark, SWE—subcritical water extract, OSE—organosolv extract, OSL—organosolv lignin, OSR—organosolv residue, PRF—polysaccharide-rich fraction. The lignin percentage represents the total amount of lignin in each fraction; KL—Klason lignin, ASL—acid-soluble lignin.

In total, 20.7% of the bark is extracted in the subcritical water extraction (100 °C—5.0%; 140 °C—8.0%; 160 °C—7.7%). To determine the amount of lignin, acid hydrolysis was performed on the extracts. The detailed carbohydrate composition of the extracts can be found in Table S2 in the Supporting Information. In Figure 6 the amount of lignin extracted in each step is shown. It was found that around 17.2% of all lignin was extracted during subcritical water extraction. Summing up the lignin from subcritical water extraction and organosolv extraction, ca. 33.3% of lignin is extracted; however, when looking at the amount remaining in the residue after the extraction sequence (37.4%), still 29.3% is missing. It is assumed that those are not precipitated from the organosolv extract and remain in the aqueous polysaccharide-rich fraction (PRF). 2D HSQC NMR analysis of the neutralized aqueous phase also confirms the presence of some lignin in the PRF, but exact determination of the mass is difficult since neutralization of the extract was performed to avoid further degradation. The 2D NMR spectrum is shown in Figure S3 in the Supporting Information; the presence of the methoxyl signal at δ_C_/δ_H_ 55.4/3.7 ppm was used as an indication. Additionally, UV/vis was performed on the aqueous phase to corroborate the presence of lignin (Figure S4 in the Supporting Information). Another hypothesis could be that the lignin content of the raw bark is overestimated. By the commonly used Klason lignin determination, it is possible that some extractives (tannin and stilbene glucoside) are condensed during the harsh acid treatment and end up in the Klason lignin, which would give the appearance of a higher lignin content than actually present in the raw bark.

Since the presence of some extractives was observed in the 100 °C fraction, this is absolutely possible. In that case, the amount of missing lignin might be much lower.

### Investigation of Residual Lignin after Organosolv Extraction

**Figure 7:**
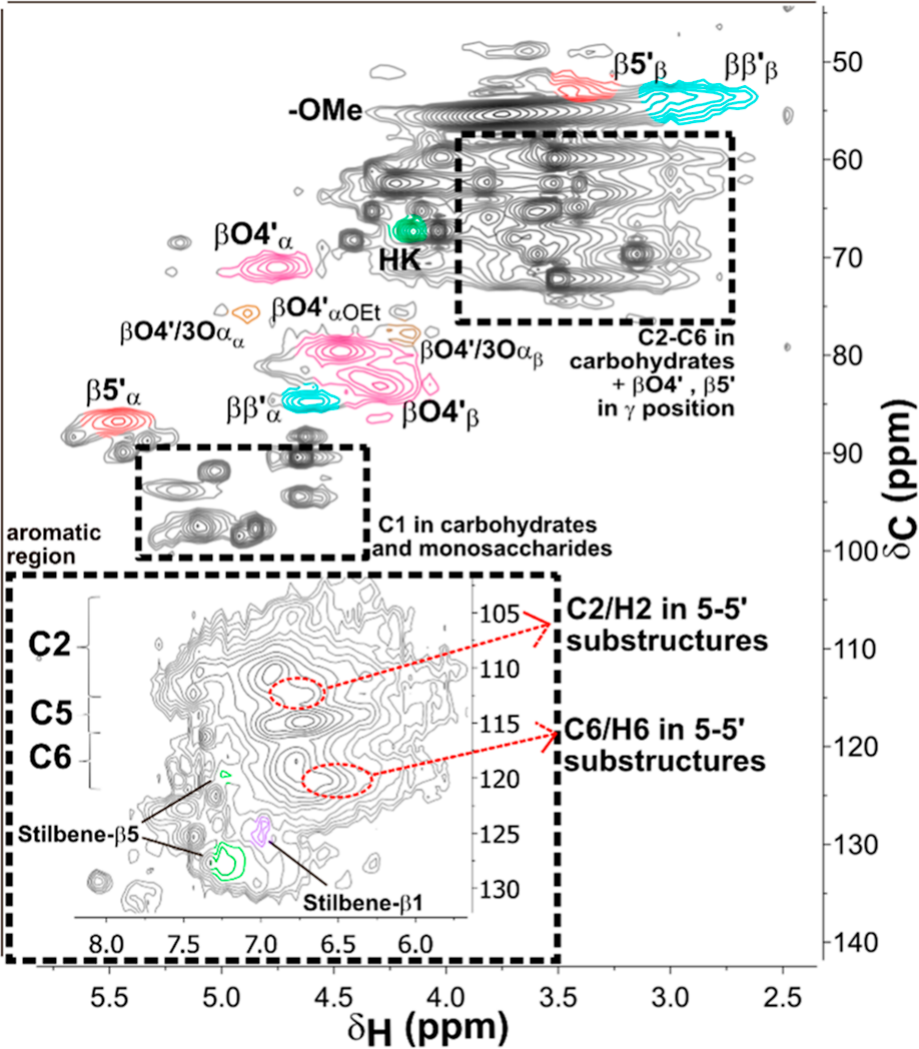
2D HSQC NMR of lignin extracted from the residue after organosolv extraction in DMSO-*d*6. The main lignin interunit linkages are assigned in the spectrum, and the associated structures are depicted in Figure 2.

Moreover, the structure of the remaining lignin, which was not extracted, was investigated to get a better understanding of the extraction process and its limitations. The residue after organosolv extraction was ball-milled and extracted with dioxane to recover the **MRL**. Analysis of the 2D HSQC NMR spectra reveals some interesting characteristics of the residual lignin (Figure 7). In the following, the chemical and physical properties of the MRL are evaluated, and possible reasons for the limited extractability of it are discussed. First of all, the amount of β-O-4′ linkages was determined by integration to be around 18%, which is significantly lower compared to the OSL and MBL (see Table 1). This observation suggests that lignin with a higher β-O-4′ content is easier to be extracted in the organosolv process, and hence the remaining lignin is poorer in β-O-4′ linkages.

It can be noticed, however, that even in the residual lignin, the β-O-4′ Cα position appears to be both hydroxylated and etherified (Figure 7). As mentioned earlier, the etherification of the β-O-4′ Cα position is assumed to improve the solubility of the lignin in ethanol. It seems that the lignin in the residue is not dissolved in ethanol, although it is etherified. The proportion of β-O-4′ Cα etherified with regard to the total amount of β-O-4′ is around 64%, which is even higher compared to the OSL. It was suspected that the MRL exhibits lower etherification, which could cause lower solubility and extractability of the lignin; however, from this result, this theory can be rejected.

It was further noticed that the signals of 5-5′ substructures are stronger compared to the OSL, which would confirm the assumption that the MRL is more condensed in its structure and therefore limits the extraction by the mild organosolv process. Lignin condensation could occur in the residual lignin since this fraction remains in the extraction vessel all through the extraction period, but it is also likely that these condensed structures are already present in the lignin of the raw bark and are enriched. Lignin condensation of the residual lignin was further investigated by ^31^P NMR quantification, and it seems that the hydroxy functionalities are similar to the OSL as can be seen in Figure 3. Other condensations might have occurred between C6 on lignin and Cα on another lignin moiety, which cannot be detected by ^31^P NMR due to the too far distance from the phenolic OH group to give a shift.

Similar to MBL in the 2D HSQC NMR spectrum, MRL signals of some polysaccharides are detected. From carbohydrate analysis it is known that the only monosaccharides found in the residue are xylose and mannose (Table S1). Thus, the presence of pectic polysaccharides can be excluded. The identity of the structures resulting in the correlation peaks around δ_C_/δ_H_ 88.0–97.5/4.6–5.2 ppm has not yet been determined and will need further investigation, but it is assumed that they are resulting from xylose and mannose structures. It is also observed that the cross-coupling peak of coniferyl alcohol and the catechol moiety of astringin at δ_C_/δ_H_ 75.8/4.90 ppm and δ_C_/δ_H_ 78.0/4.15 ppm is still present in the residue, but the 8-O-4′coupling of isorhapontin and astringin at around δ_C_/δ_H_ 79.0/5.0 ppm is not detected anymore. Their integration into the lignin structure might also be a reason for the limited extractability of the bark lignin. It can be some indication for limited accessibility of the solvent in the organosolv extraction, which is overcome in the dioxane extraction by the ball milling of the sample.

Finally, the molecular weight of the lignin plays an important role when it comes to solubility. In Figure 4, the molecular weights of OSL, MBL, and MRL are compared. It can be clearly seen that the PD of the MRL is significantly higher compared to OSL and MBL, but with regard to the molecular weight, no distinct conclusion can be drawn. Although the weight-average molecular weight of MRL is considerably higher, the number-average molecular weight of all three lignins is not significantly different. Although it was expected to find a significantly higher molecular weight of the MRL and MBL, this was not confirmed by SEC analysis. An important thing to consider is the ball milling of the sample, which also might induce some degradation and cleavage of bonds that could result in lower molecular weight of MBL and MRL.

In conclusion, the most plausible factor that reduces the extractability of the lignin from the bark could be the limited accessibility of the lignin by the solvent and possibly condensation reactions. Parameters such as higher molecular weight were not confirmed by the methods used in this study.

## Conclusions

Extraction of lignin from Norway spruce bark will be an interesting process for the development of future bio-based materials. The use of a cyclic organosolv process allows for the extraction of high-quality lignin with promising properties. The high β-O-4′ content, low condensation, and favored low PD facilitates the application of lignin in direct development processes of polymer precursors. Incorporated structures such as hydroxystilbene glucosides arising from hydroxystilbene glucoside moieties originally incorporated into the lignin of Norway spruce bark add antimicrobial and antioxidant properties, which further increase the interest into this precious bark lignin type. The integrated biorefinery enables the recovery of pectic polysaccharides and extractives from the bark, which have already been investigated to show interesting properties. In general, bio-based materials from the bark, an inexpensive raw material generated as a side product of the pulp and paper industry, can reduce the environmental impact created by non-bio-based materials. While the raw material is inexpensive, technical feasibility and sustainability of the process are critical and remain to be assessed.

## Supporting Information Available

Chemical composition of the raw bark and the residue after organosolv extraction (OSR), chemical composition of the subcritical water extracts, 2D HSQC NMR of MBL from Norway spruce inner bark, 2D HSQC NMR of subcritical water extracts, 2D HSQC NMR of the polysaccharide part of the organosolv extracts, UV/vis spectrum of PRF, and reaction scheme on the formation of stilbene structures (PDF)
