# Supplementary material for: Fundamental Insights on the Physical and Chemical Properties of Organosolv Lignin from Norway Spruce Bark
Source: Biomacromolecules. 2022 Jul 11;23(8):3349–58. doi: 10.1021/acs.biomac.2c00457 (PMC9364314; doi:10.1021/acs.biomac.2c00457)
Supplement: Supplementary file 1 — bm2c00457_si_001.pdf [file bm2c00457_si_001.pdf]

Supporting Information

Fundamental insights on the physical and chemical properties of organosolv lignin from Norway spruce bark

Barbara Rietzler, Maria Karlsson, Isabella Kwan, Martin Lawoko, and Monica Ek*

Division of Wood Chemistry and Pulp Technology, Department Fibre and Polymer Technology and Wallenberg Wood Science Centre (WWSC), School of Engineering Sciences in Chemistry, Biotechnology and Health (CBH), KTH Royal Institute of Technology, Teknikringen 56, SE-100 44 Stockholm, Sweden

*Corresponding author: Martin Lawoko, lawoko@kth.se

Number of pages: 5

Number of figures: 4

Number of tables: 2

Number of schemes: 1

Table S1. Chemical composition of the raw bark and the residue after organosolv extraction (OSR) in % w/w and the yield of OSR in % w/w based on the initial mass of raw bark. The results are the average of at least 3 determinations and the corresponding standard deviation

component	raw bark	residue after organosolv extraction
Yield		29.2 ± 1.6
arabinose	4.5 ± 0.03	< 0.1
rhamnose	0.7 ± 0.03	< 0.1
galactose	1.9 ±0.03	< 0.1
glucose	23.7 ± 0.7	63.9 ± 3.2
xylose	4.5 ± 0.4	1.3 ± 0.4
mannose	2.3 ± 0.2	0.8 ± 0.3
galacturonic acid	9.5 ± 0.6	< 0.1
glucuronic acid	0.2 ± 0.07	< 0.1
Klason Lignin	19.2 ± 0.4	24.8 ± 0.7
acid soluble lignin	1.9 ± 0.3	2.2 ± 1.2
extractives	20.6 ± 1.1	n.d.
other	11.1 ± 3.8	7.0 ± 5.8

Table S2. Chemical composition of the subcritical water extracts at 100, 140 and 160˚C in % w/w and the yield in % w/w based on the initial mass of raw bark. The results are the average of at least 3 determinations and the corresponding standard deviation

component	100˚C	140˚C	160˚C
Yield	5.0 ± 0.3	8.0 ± 0.3	7.7 ± 2.0
arabinose	5.1 ± 1.0	26.8 ± 2.3	19.2 ± 0.2
rhamnose	1.2 ± 0.1	3.8 ± 0.1	3.2 ± 0.1
galactose	5.0 ± 1.9	5.2 ± 0.8	5.8 ± 1.4
glucose	12.1 ± 2.0	5.2 ± 1.7	6.5 ± 1.1
xylose	0.6 ± 0.5	1.7 ± 0.6	7.9 ± 2.2
mannose	1.5 ± 0.1	2.6 ± 0.1	4.1 ± 1.0
galacturonic acid	7.6 ± 0.6	26.1 ± 3.1	10.6 ± 1.6
glucuronic acid	0.8 ± 0.1	0.4 ± 0.2	0.4 ± 0.2
Klason Lignin	21.3 ± 3.4	11.2 ± 0.7	11.7 ± 4.0
acid soluble lignin	3.9 ± 0.4	3.4 ± 0.7	3.7 ± 1.0

**other**	40.8 ± 3.0	13.6 ± 6.8	26.9 ± 4.5

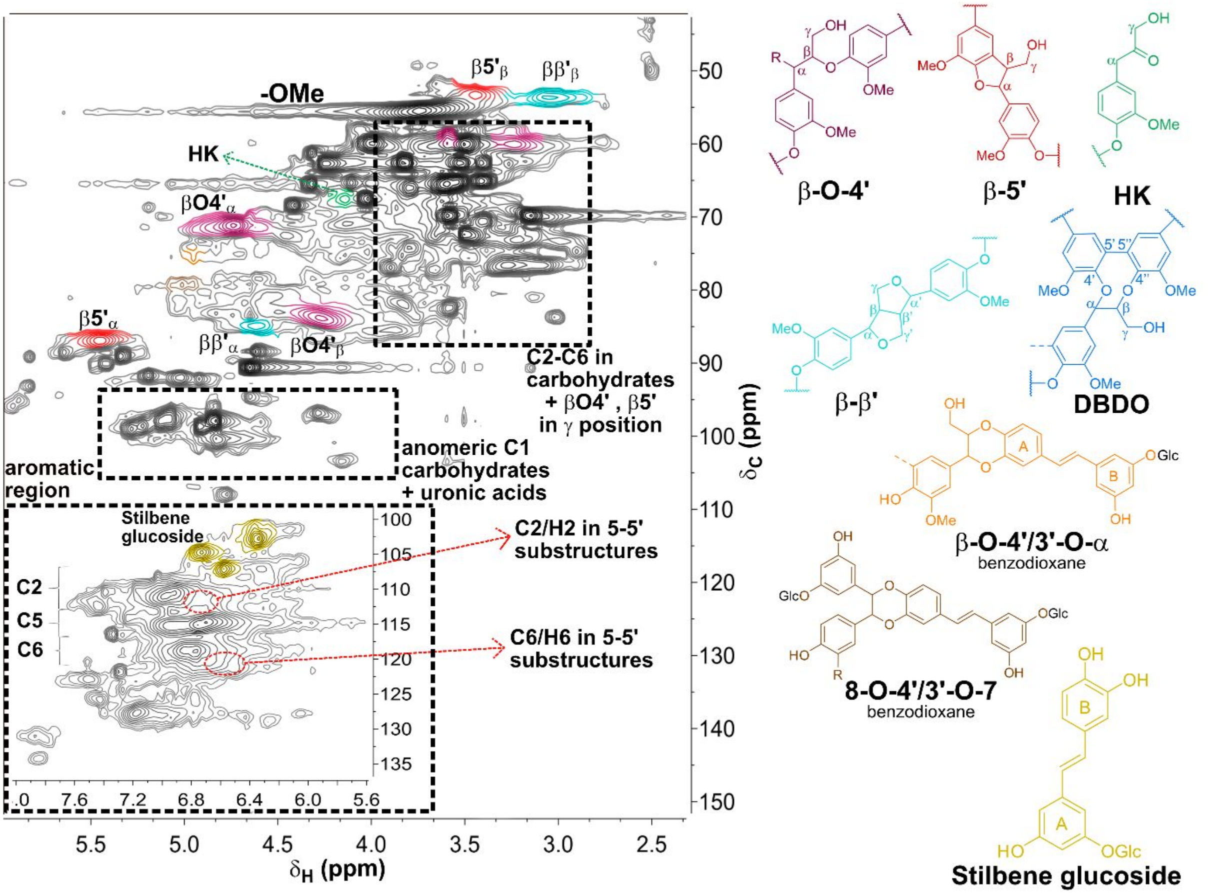

Figure S1. 2D HSQC NMR of milled bark lignin (MBL) from Norway spruce inner bark in dimethyl sulfoxide-*d6*. The main lignin inter-unit linkages are depicted to the right of the spectrum. β-O-4' aryl ethers; β-5' phenylcoumarans; β-β' resinols; 5-5' dibenzodioxocins (DBDO); Hibbert's ketone (HK); β-O-4'/3-O-α benzodioxane structure formed through coupling of coniferyl alcohol and astringin; 8-O-4'/3'-O-7 benzodioxane structure formed by stilbene glucoside units; Stilbene glucoside

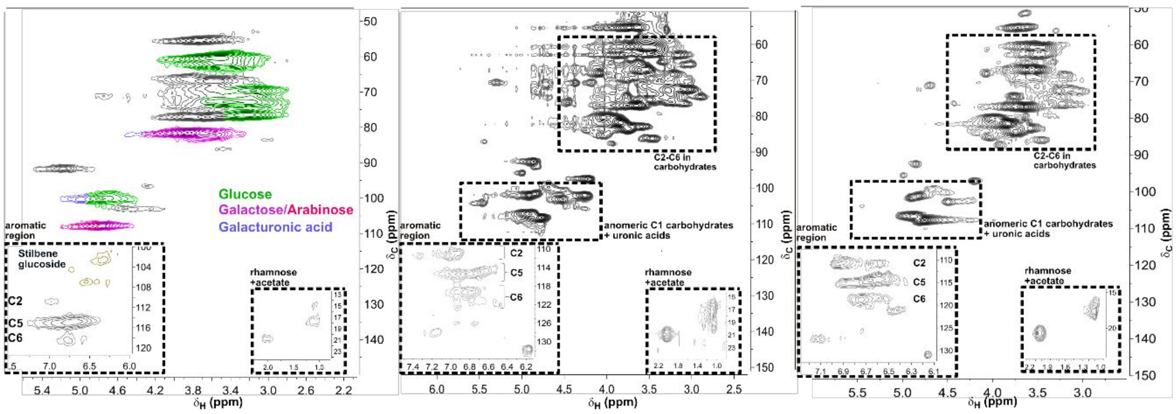

Figure S2. 2D HSQC NMR of subcritical water extracts at 100, 140 and 160˚C in dimethyl sulfoxide-*d6*

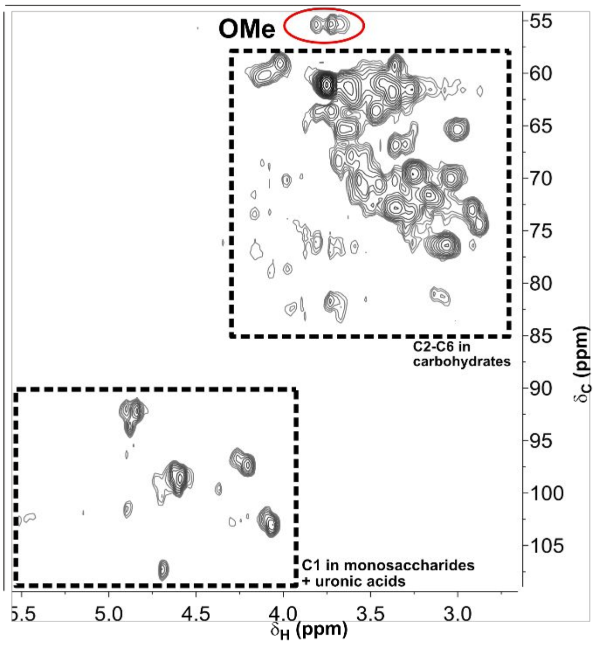

Figure S3. 2D HSQC NMR of polysaccharide-rich fraction after organosolv extraction in dimethyl sulfoxide-*d6*

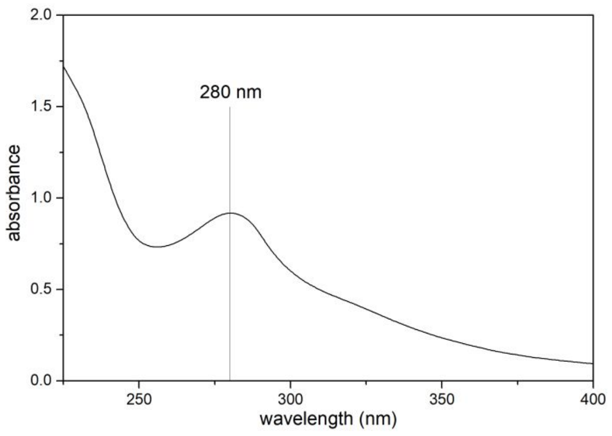

Figure S4. UV/Vis spectrum of polysaccharide-rich fraction after organosolv extraction between 225 nm and 400 nm

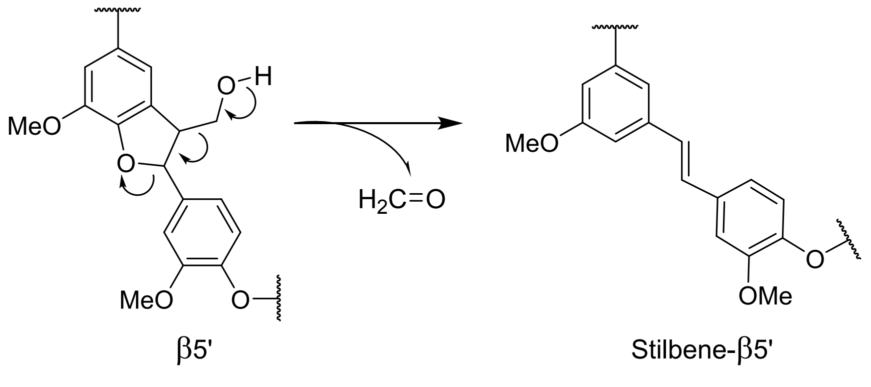

Scheme S1. Formation of stilbene-β1’ and stilbene-β5’ structures through elimination of formaldehyde
